# School-based predictive contributors to six-minute walk test performance across pubertal stages: an explainable machine learning study in Tunisian adolescents

**DOI:** 10.3389/fspor.2026.1809681

**Published:** 2026-09-18

**Authors:** Hatem Ghouili, Amel Dridi, Nejmeddine Ouerghi, Jaouher Hamaidi, Wissem Dhahbi, Halil İbrahim Ceylan, Valentina Stefanica, Raul Ioan Muntean, Ismail Dergaa, Anissa Bouassida, Noomen Guelmami

**Affiliations:** 1 Research Unit: Sports Science, Health and Movement, High Institute of Sport and Physical Education of Kef, University of Jendouba, El Kef, Tunisia; 2 Physical Education and Sports Teaching Department, Faculty of Sports Sciences, Atatürk University, Erzurum, Türkiye; 3 Department of Physical Education and Sport, Faculty of Sciences, Physical Education and Informatics, National University of Science and Technology Politehnica Bucharest, Pitești University Center, Pitești, Romania; 4 Department of Physical Education and Sport, Faculty of Law and Social Sciences, University “1 Decembrie 1918” of Alba Iulia, Alba Iulia, Romania; 5 Higher Institute of Sport and Physical Education of Ksar Saïd, University of Manouba, Manouba, Tunisia; 6 Research Unit Physical Activity, Sport and Health (UR18JS01), National Observatory of Sport, Tunis, Tunisia

**Keywords:** adolescence, biological maturation, cardiovascular fitness, clustering analysis, explainable machine learning, functional capacity, peak height velocity, physical fitness

## Abstract

**Background:**

The six-minute walk test (6MWT) provides a practical measure of submaximal functional exercise capacity in adolescents and reflects the integrated response of cardiovascular, respiratory, and musculoskeletal systems during walking. Traditional age-based linear models inadequately capture nonlinear, maturation-dependent interactions among anthropometric, behavioral, and physiological factors. This study aimed to (i) identify stage-specific predictive contributors to 6MWT performance using supervised machine learning, (ii) improve interpretability through explainable artificial intelligence, and (iii) explore data-driven functional and cardiovascular profiles across peak height velocity (PHV) stages.

**Methods:**

In this cross-sectional school-based study, 3,166 Tunisian adolescents (11–18 years) were categorized as pre-PHV (*n* = 959), around-PHV (*n* = 953), or post-PHV (*n* = 1,254). Submaximal functional exercise capacity was assessed using the 6MWT distance. Random forest regression models were developed globally and stratified by maturation stage. Predictive contributors were interpreted using permutation importance and Shapley Additive Explanations (SHAP). Exploratory k-means clustering based only on 6MWT and cardiovascular response variables was used to identify data-driven profiles within each PHV stage.

**Results:**

The global model explained 40% of the variance in 6MWT distance (R^2^ = 0.399; RMSE = 121 m). Predictive performance varied markedly by maturation stage, with low explanatory power pre-PHV (R^2^ = 0.039), intermediate performance around-PHV (R^2^ = 0.178), and higher predictability post-PHV (R^2^ = 0.347). Physical activity was the highest-ranking predictor globally and across all PHV stages, followed by chronological age, particularly around-PHV. SHAP analyses showed progressively larger mean absolute contributions of physical activity across PHV stages, reaching approximately 45 m in post-PHV participants. Although the gap statistic favored a one-cluster solution, exploratory global clustering with k = 2 described a lower-response profile [6MWT distance = 490.4 ± 132.1 m; exercise-induced heart rate response (ΔHR) = 68.4 ± 12.6 bpm] and a higher-response profile (6MWT distance = 599.0 ± 157.1 m; ΔHR = 91.3 ± 13.3 bpm), both of which were represented across all PHV stages.

**Conclusions:**

In this cross-sectional cohort, 6MWT performance showed maturation-stage-specific prediction patterns, but model performance remained modest, particularly before PHV. Physical activity had the strongest model contribution after PHV and may represent an important modifiable correlate, although causal inference cannot be made from these data. The exploratory functional and cardiovascular profiles should be interpreted as data-driven clusters rather than stable biological states. These findings support maturation-sensitive assessment of adolescent 6MWT performance and highlight the need for longitudinal and interventional studies.

## 1 Introduction

Submaximal functional exercise capacity during adolescence is an important determinant of current and future health, reflecting the integrated function of the cardiovascular, respiratory, and musculoskeletal systems during sustained activity (1). The six-minute walk test (6MWT) is commonly used to assess this construct in field and clinical settings, and the total distance covered provides a practical marker of submaximal walking performance rather than a complete measure of all dimensions of functional capacity (2–4). In pediatric populations, lower functional capacity has been linked to adverse cardiovascular risk profiles and an increased risk of chronic diseases in adulthood (5–7). Despite its clinical and public health relevance, the contributors to 6MWT performance during adolescence remain incompletely understood, particularly in relation to biological maturation.

Adolescence is characterized by substantial inter-individual heterogeneity driven by pubertal development. Chronological age poorly captures this variability, as adolescents of the same age may differ by several years in biological maturation (7–9). Puberty involves rapid somatic growth, hormonal changes, and structural adaptations that influence skeletal muscle mass, cardiovascular dimensions, and respiratory capacity (10–15). Peak height velocity (PHV), defined as the period of maximal growth rate, represents a key maturational milestone and provides a more biologically meaningful indicator of development than chronological age alone (8, 16). The timing and tempo of PHV vary widely among individuals, resulting in pronounced differences in body size, body proportions, cardiovascular responses, and functional performance (17–19). During the peri-pubertal period, rapid and asynchronous growth of body segments may further affect biomechanics and movement efficiency (20, 21). These maturational processes substantially influence functional capacity but are rarely explicitly accounted for in analytical models.

Previous studies on the functional abilities of young people have largely relied on traditional statistical approaches, such as linear regression and age-group comparisons (22–24). While informative, these methods assume linear relationships and often fail to capture the complex, nonlinear interactions among the anthropometric, physiological, behavioral, and maturational factors that characterise adolescence (24, 25). Many studies treat adolescent populations as homogeneous groups, potentially obscuring maturation-specific prediction patterns and overlooking determinants that may contribute differently before, during, and after puberty (26, 27). As a result, the relative predictive importance of modifiable behaviors, body size and composition, and cardiovascular responses at different pubertal stages remains poorly defined.

Machine learning approaches provide a robust framework to address these limitations. Tree-based algorithms, such as random forest (RF) regression, can model nonlinear associations, handle high-dimensional predictor sets, and capture higher-order interactions without restrictive parametric assumptions (28, 29). The application of machine learning in pediatric and clinical research has shown improved predictive performance compared to traditional regression methods, particularly in heterogeneous populations (30, 31). However, most machine learning studies prioritize prediction accuracy over a deeper understanding of the underlying mechanisms, thus limiting their translational value (32).

Explainable machine learning techniques address this limitation by providing a transparent interpretation of complex models. Permutation-based importance measures quantify the relative contribution of predictors to model performance, while Shapley Additive Explanations (SHAP) decompose individual predictions into variable-specific contributions, expressed in meaningful units (33, 34). These approaches enable the identification of dominant determinants, the characterization of nonlinear relationships, and the comparison of predictor effects across subgroups. Despite their increasing use in medical research (30, 33, 35), explainable machine learning methods remain underutilized in developmental exercise science, particularly in studies explicitly addressing biological maturation.

Beyond identifying model-based contributors to 6MWT performance, unsupervised machine learning approaches can reveal further exploratory insights by revealing data-driven functional profiles. Clustering methods have been used to identify vulnerable and resilient fitness profiles in adult populations (36–38), but their application to adolescent 6MWT performance remains limited. Because the present study is cross-sectional, it cannot evaluate within-individual stability of profiles during pubertal development; it can only examine whether comparable profiles and their distribution are observed across PHV stages. Identifying adolescents with lower functional and cardiovascular response profiles during critical developmental periods may help generate hypotheses for future longitudinal and intervention studies.

Therefore, the present study aimed to address these gaps by integrating supervised and unsupervised machine learning approaches within a maturation-specific, cross-sectional framework. Specifically, this study sought to: (i) identify predictive contributors to 6MWT distance within pre-PHV, around-PHV, and post-PHV stages using stage-stratified random forest regression models based on pubertal growth velocity (PHV); (ii) enhance the interpretability of model predictions through explainable machine learning techniques; and (iii) identify exploratory functional and cardiovascular profiles based solely on functional and cardiovascular variables, and examine their distribution and physiological characteristics across PHV stages.

## 2 Materials and methods

### 2.1 Ethical approval

This study was conducted in accordance with the Declaration of Helsinki. The research protocol was reviewed and approved by the Institutional Scientific and Ethical Committee of Physical Education and Sport at the University of Jendouba, Tunisia (reference: PHS-31/2022). The study procedures complied with the relevant requirements and procedures governing research in sports medicine and exercise science (39).

### 2.2 Sample size calculation

Sample size calculation was designed to ensure adequate statistical power for multivariate modeling, machine learning analyses, and assessments stratified by biological maturation. For observational studies evaluating functional and physiological outcomes in adolescents, the minimum sample size was estimated using the formula *n* = Z^2^p(1-p)/d^2^, where Z = 1.96 (standard normal variable for 95% confidence level), *p* = 0.15 (expected proportion), and d = 0.02 (desired precision). This yielded a required minimum sample of 1,224 participants. Established recommendations indicate that samples exceeding 1,000 observations provide sufficient statistical power to detect small to medium effects and ensure stable parameter estimates in regression and tree-based models (40). Machine learning models, including random forests, demonstrate reliable performance and accurate variable importance estimates when sample sizes exceed several hundred observations per analytical stratum (41). Previous large-scale studies examining functional and behavioral determinants in adolescents typically included samples ranging from 2,000 to over 5,000 participants (42, 43). The final sample in this study substantially exceeded calculated minimum requirements, providing sufficient statistical power for supervised learning, unsupervised profiling, explainable artificial intelligence analyses, and studies stratified by biological maturation stage.

### 2.3 Participants

Participants were recruited from nine Tunisian schools, including two primary schools (grades 4–5), four middle schools, and three high schools, ensuring coverage of early, middle, and late adolescence. Recruitment was conducted in collaboration with school administrations and physical education departments, and all assessments were performed during school hours. Eligibility criteria included: (i) adolescent age range corresponding to compulsory school education (approximately 11–18 years); (ii) current school enrollment at the time of data collection; and (iii) availability of complete data for anthropometric measurements, functional performance indicators, cardiovascular parameters, and questionnaire variables. Exclusion criteria were: (i) diagnosed endocrine or metabolic disorders known to affect growth, biological maturation, or cardiovascular regulation; (ii) chronic medication use with potential influence on cardiovascular or metabolic responses; (iii) musculoskeletal, neurological, or medical conditions precluding safe participation in functional testing; and (iv) incomplete or missing data for any required variable.

Of the 3,428 adolescents initially screened, 3,166 met all eligibility criteria and were included in the final analyses. Exclusions were due to incomplete anthropometric, cardiovascular, functional, or questionnaire data; absence or withdrawal during the assessment session; missing consent or assent; or health/safety criteria that contraindicated participation in the 6MWT. All measurements were conducted by trained and certified investigators specialized in anthropometry, cardiovascular assessment, and functional performance testing.

### 2.4 Experimental design

This cross-sectional study was conducted during school hours under standardized conditions. All assessments occurred in dedicated rooms at participating schools to ensure confidentiality and measurement consistency. To minimize circadian variation influences, anthropometric and functional assessments were conducted between 08:00 and 11:00 h (44, 45). Participants wore light indoor clothing and were barefoot during measurements. Body mass was measured to the nearest 0.1 kg using a calibrated electronic scale (Seca 813, Hamburg, Germany), and standing height was measured to the nearest 0.1 cm using a portable height measuring device (Seca 213, Hamburg, Germany), following standardized anthropometric protocols (46). Seated height was measured with participants sitting upright on standardized measuring tables, torso vertically aligned, and feet flat on the floor. Leg length was calculated as the difference between standing and seated height. Waist circumference was measured midway between the lower edge of the last rib and the iliac crest, at the end of normal expiration, using a non-elastic measuring tape (47). Body mass index (BMI) was calculated by dividing body mass (kg) by the square of height (m^2^). Because of the large sample size and the restricted time available for school-based assessments, anthropometric measurements were obtained once per participant and were not repeated. To minimize measurement error, all measurements were performed by trained investigators using calibrated equipment under standardized conditions and under the direct supervision of senior researchers.

**Table 1:** Participant characteristics across PHV stages.

Characteristic	PHV stage			*p*-value^b^
	Pre-PHV	Around-PHV	Post-PHV	
	*N* = 959^a^	*N* = 953^a^	*N* = 1,254^a^	
Age (years)	12.92 ± 0.73	15.38 ± 1.11	17.00 ± 1.48	<0.001
Sex				0.420
Male	545 (57%)	554 (58%)	694 (55%)	
Female	414 (43%)	399 (42%)	560 (45%)	
Height (cm)	156.16 ± 8.87	177.55 ± 4.11	180.93 ± 7.86	<0.001
Weight (kg)	49.87 ± 7.14	66.02 ± 10.36	75.39 ± 13.59	<0.001
BMI (kg/m^2^)	20.43 ± 2.10	20.91 ± 2.91	22.93 ± 3.25	<0.001
WC (cm)	78.73 ± 6.94	82.67 ± 9.41	84.05 ± 11.32	<0.001
Physical activity score	2.85 ± 1.05	3.08 ± 1.08	2.73 ± 1.09	<0.001
Sleep duration (h/night)	8.59 ± 0.94	8.23 ± 0.92	7.99 ± 1.00	<0.001
Diet diversity score	4.69 ± 2.85	5.19 ± 2.89	5.46 ± 2.93	<0.001
PSS-10 score	18.54 ± 3.66	17.52 ± 3.76	16.80 ± 3.80	<0.001
6MWT distance (m)	452.02 ± 114.21	555.73 ± 134.27	620.97 ± 159.42	<0.001
ΔHR (bpm)	77.80 ± 17.70	81.70 ± 16.68	82.91 ± 17.14	<0.001

a Mean ± SD; *n* (%).

b One-way ANOVA for continuous variables; Pearson chi-square test for sex.

### 2.5 Functional capacity and physiological assessment

#### 2.5.1 Six-minute walk test

Submaximal functional exercise capacity was assessed using the 6MWT, administered according to the American Thoracic Society's standardized guidelines (1). The total distance covered (meters) was used as the primary performance outcome. The 6MWT was therefore interpreted as an indicator of submaximal functional exercise capacity and walking performance, not as a complete multidimensional assessment of functional capacity.

#### 2.5.2 Heart rate assessment

Heart rate was measured using the Polar Team Sport system (Polar Electro OY, Kempele, Finland). Resting heart rate (RHR) was assessed in a seated position before test initiation, after a minimum of five minutes’ rest. Heart rate was recorded continuously throughout the test until completion. Peak heart rate (HR_peak) represented the highest value observed during the test. Heart rate response to exercise was calculated using the following formula:

ΔHR=HR_peak–RHR

where ΔHR represents heart rate response, HR_peak is the maximal heart rate observed during the 6MWT, and RHR is the resting heart rate measured before the test.

### 2.6 Assessment of lifestyle and psychosocial factors

Physical activity was assessed using the Physical Activity Questionnaire for Older Children (PAQ-C) for participants aged 11–13 years and the Physical Activity Questionnaire for Adolescents (PAQ-A) for those aged 14–18 years (48, 49). Both instruments assess usual moderate-to-vigorous physical activity over the previous 7 days across various settings and provide a composite score from 1 to 5, with higher scores indicating greater activity levels. Sleep duration was estimated from participants’ usual bedtimes and wake-up times on weekdays and weekends. A weighted average nighttime sleep duration was calculated using the formula (weekday  ×   5 + weekend  ×   2)/7, expressed in hours per night (50). Perceived psychosocial stress was assessed using the 10-item Perceived Stress Scale (PSS-10), which measures the extent to which individuals perceive life situations as unpredictable, uncontrollable, and stressful during the previous month (51). Items were rated on a 5-point Likert scale, with total scores ranging from 0 to 40; higher scores indicated greater perceived stress. Dietary diversity was assessed using a food-group-based dietary diversity score derived from the Food and Agriculture Organization recommendations (52). Participants reported consumption of predefined food groups over the previous 24 h, with one point awarded for each food group consumed.

### 2.7 Biological maturation assessment

Biological maturation was estimated using Mirwald maturity offset equations, which are sex-specific and derived from anthropometric measurements including chronological age, standing height, sitting height, leg length, and body mass (53). These equations estimate the number of years before (negative values) or after (positive values) PHV. Based on maturity offset values, participants were classified into three biologically relevant maturation stages: pre-PHV (<−1.0 year), around-PHV (−1.0 to +1.0 year), and post-PHV (>+1.0 year). This categorization allowed analyses to explicitly account for maturation-related heterogeneity in growth, cardiovascular responses, and functional performance.

### 2.8 Statistical analysis and machine learning framework

All statistical and machine learning analyses were conducted using R software (version 4.3.x; R Foundation for Statistical Computing, Vienna, Austria). Statistical significance was set at *p* < 0.05 for all inferential analyses. Descriptive statistics are reported as means with standard deviations for continuous variables and as counts with percentages for categorical variables. Distribution of continuous variables was assessed using the Shapiro–Wilk test. Comparisons between stages of biological maturation (pre-PHV, around-PHV, and post-PHV) were performed using one-way analysis of variance (ANOVA) with Tukey's *post hoc* test for continuous variables and the chi-square test for categorical variables. Effect sizes were quantified using eta squared (*η*^2^).

Supervised machine learning analyses were conducted to predict 6MWT distance, which was modeled as a continuous outcome. RF regression was selected for its ability to accommodate nonlinear relationships, correlated predictors, and higher-order interactions (28, 29). RF models were fitted using the global dataset and separately within each PHV stage to capture maturation-specific associations. Predictor variables included anthropometric measures, biological maturation indicators, cardiovascular parameters, lifestyle behaviors, and psychosocial factors. Sex was entered as a binary-coded predictor, whereas PHV stage was included only in the global model as a categorical predictor. To reduce structural redundancy among anthropometric predictors, sitting height and leg length were excluded, whereas standing height was retained as an interpretable overall indicator of somatic growth. Model performance was assessed across five repeated random 80/20 splits between training and test data, providing an independent estimate of predictive accuracy. Final models used for permutation-importance and SHAP analyses were refitted on the complete corresponding datasets using 2,000 trees. Predictive performance was summarized using root mean squared error (RMSE), mean absolute error (MAE), and coefficient of determination (R2), both globally and according to PHV stage.

To improve interpretability, explainable machine learning techniques were applied. Variable importance was quantified using permutation-based importance measures derived from trained RF models, computed for the global model and within each PHV stage. SHAP values were used to estimate the model-based contribution of each predictor to individual 6MWT distance predictions while accounting for nonlinear relationships and feature interactions (33, 54). For each participant, SHAP values represent the contribution of a predictor, expressed in meters, relative to the model's baseline prediction. Mean absolute SHAP values were used to summarize the overall magnitude of each predictor's contribution. SHAP beeswarm plots illustrated the direction and distribution of individual contributions.

Unsupervised machine learning was applied to explore functional-cardiovascular profiles independently of anthropometric, maturational, lifestyle, and psychosocial variables. K-means clustering was performed on standardized functional and cardiovascular variables only, including 6MWT distance, RHR, and exercise-induced heart rate response (ΔHR). No anthropometric, maturational, lifestyle, or psychosocial variables were included in the clustering procedure. Clustering was performed once in the complete sample. The resulting global profile assignments were subsequently described and visualized separately according to PHV stage; no independent clustering analysis was conducted within each maturation group.

The number of clusters was evaluated using the gap statistic and average silhouette width for solutions ranging from k = 1 to k = 6 (55). As the gap statistic did not support a strong natural clustering structure beyond k = 1, no assumption of naturally discrete subgroups was made. For exploratory descriptive purposes, k = 2 was retained as the first non-trivial solution because it showed the highest average silhouette width among the multi-cluster solutions examined and produced physiologically interpretable patterns. This two-cluster solution was not considered evidence of distinct or stable biological states. Clusters were labeled a posteriori as lower-response and higher-response functional-cardiovascular profiles according to their centroid patterns in 6MWT distance, resting heart rate, and exercise-induced heart rate response (ΔHR). Principal component analysis (PCA) was applied to standardized functional variables for visualization purposes only (56). PCA was performed once in the complete sample, and the resulting participant scores and global profile assignments were subsequently displayed according to PHV stage. Following cluster identification, profiles were characterized using cluster centroids and descriptive statistics for 6MWT distance, RHR, and ΔHR, both globally and within each PHV stage.

### 2.9 Use of artificial intelligence in manuscript preparation

In preparing this manuscript, the authors used the ChatGPT language model (OpenAI, San Francisco, CA, USA) solely for linguistic correction and grammatical improvement (57). The study design, data collection, statistical analyses, interpretation of results, and formulation of conclusions were carried out entirely by the authors, who assume full responsibility for the scientific integrity and content of the manuscript.

## 3 Results

### 3.1 Participant characteristics across peak height velocity stages

Table 1 presents demographic, anthropometric, lifestyle, psychosocial, and functional characteristics of participants stratified by biological maturation stage. Chronological age differed markedly across PHV stages (*p* < 0.001), with post-PHV participants being the oldest (17.00 ± 1.48 years), followed by around-PHV (15.38 ± 1.11 years), while pre-PHV adolescents were the youngest (12.92 ± 0.73 years). Sex distribution did not differ significantly across PHV stages (*p* = 0.420).

All anthropometric variables showed highly significant differences across PHV stages (*p* < 0.001). Standing height increased progressively from pre-PHV (156.16 ± 8.87 cm) to around-PHV (177.55 ± 4.11 cm), reaching the highest values in post-PHV adolescents (180.93 ± 7.86 cm). Body mass, BMI, and waist circumference also differed significantly across PHV stages (all *p* < 0.001).              

Lifestyle behaviors differed significantly across PHV stages (*p* < 0.001). Physical activity scores differed significantly across PHV stages but did not show a monotonic increase. The highest mean score was observed in the around-PHV group (3.08 ± 1.08), followed by pre-PHV (2.85 ± 1.05) and post-PHV participants (2.73 ± 1.09; *p* < 0.001). Sleep duration decreased across PHV stages, whereas dietary diversity increased and perceived stress decreased (all *p* < 0.001).

Significant differences in functional performance were observed across PHV stages (*p* < 0.001). 6MWT distance increased substantially with maturation, from 452.02 ± 114.21 m in pre-PHV adolescents to 555.73 ± 134.27 m in around-PHV, reaching the highest values in post-PHV participants (620.97 ± 159.42 m). Heart rate response to exercise (ΔHR) was significantly greater in post-PHV adolescents (82.91 ± 17.14 bpm) compared with around-PHV (81.70 ± 16.68 bpm) and pre-PHV groups (77.80 ± 17.70 bpm).

### 3.2 Supervised machine learning performance across peak height velocity stages

Table 2 presents the predictive performance of RF models for the 6MWT distance. At the global level, the model demonstrated moderate explanatory capacity, accounting for approximately 40% of the variance in functional performance (R^2^ = 0.399), with an RMSE of 121 m and an MAE of 97.2 m.

**Table 2:** Predictive performance of random forest models for 6MWT distance.

Model scope	PHV stage	N	RMSE (m)	MAE (m)	R^2^
Global model	All participants	3,166	121.0 ± 0.1	97.2 ± 0.1	0.399 ± 0.001
PHV-stratified model	Pre-PHV	959	111.9 ± 0.4	91.0 ± 0.4	0.039 ± 0.007
	Around-PHV	953	121.7 ± 0.6	97.3 ± 0.4	0.178 ± 0.009
	Post-PHV	1,254	128.8 ± 0.4	103.7 ± 0.3	0.347 ± 0.004

Values are presented as mean ± SD across five repeated random 80/20 training–test splits. RMSE, MAE, and R^2^ were calculated for each repetition and subsequently summarized across the five repetitions. RMSE, root mean squared error; MAE, mean absolute error; R^2^, coefficient of determination; PHV, peak height velocity.

Stratified analyses revealed marked differences in model performance across PHV stages. In the pre-PHV group, explanatory power was minimal (R^2^ = 0.039), despite relatively lower prediction errors (RMSE = 111.9 m; MAE = 91.0 m). This finding suggests substantial unexplained interindividual variability at early maturation stages, likely reflecting weak coupling between chronological age and biological development before PHV. The model performed substantially better in the post-PHV group (R^2^ = 0.347), albeit with higher absolute prediction errors (RMSE = 128.8 m; MAE = 103.7 m), consistent with wider dispersion of functional performance following pubertal maturation. Adolescents around-PHV exhibited intermediate predictive performance (R^2^ = 0.178), reflecting pronounced biological heterogeneity characteristic of the pubertal growth spurt. Overall, these results indicate that the predictability of functional capacity is strongly dependent on biological maturation stage and that global models may obscure stage-specific determinants and performance patterns.

### 3.3 Global and PHV stage-specific predictive contributions to 6MWT performance: permutation importance analysis

Figure 1 presents normalized permutation-based variable importance (0-1) derived from optimal RF models, estimated globally and separately for each PHV stage. In the global model, physical activity showed the highest relative predictive contribution to 6MWT distance and was therefore assigned a normalized importance of 1.00. Chronological age ranked second (0.47), followed by BMI (0.27), standing height (0.26), body mass (0.26), waist circumference (0.22), and PHV stage (0.18). Resting heart rate, sex, exercise-induced heart rate response, and perceived stress showed comparatively smaller contributions.  

**Figure 1:**
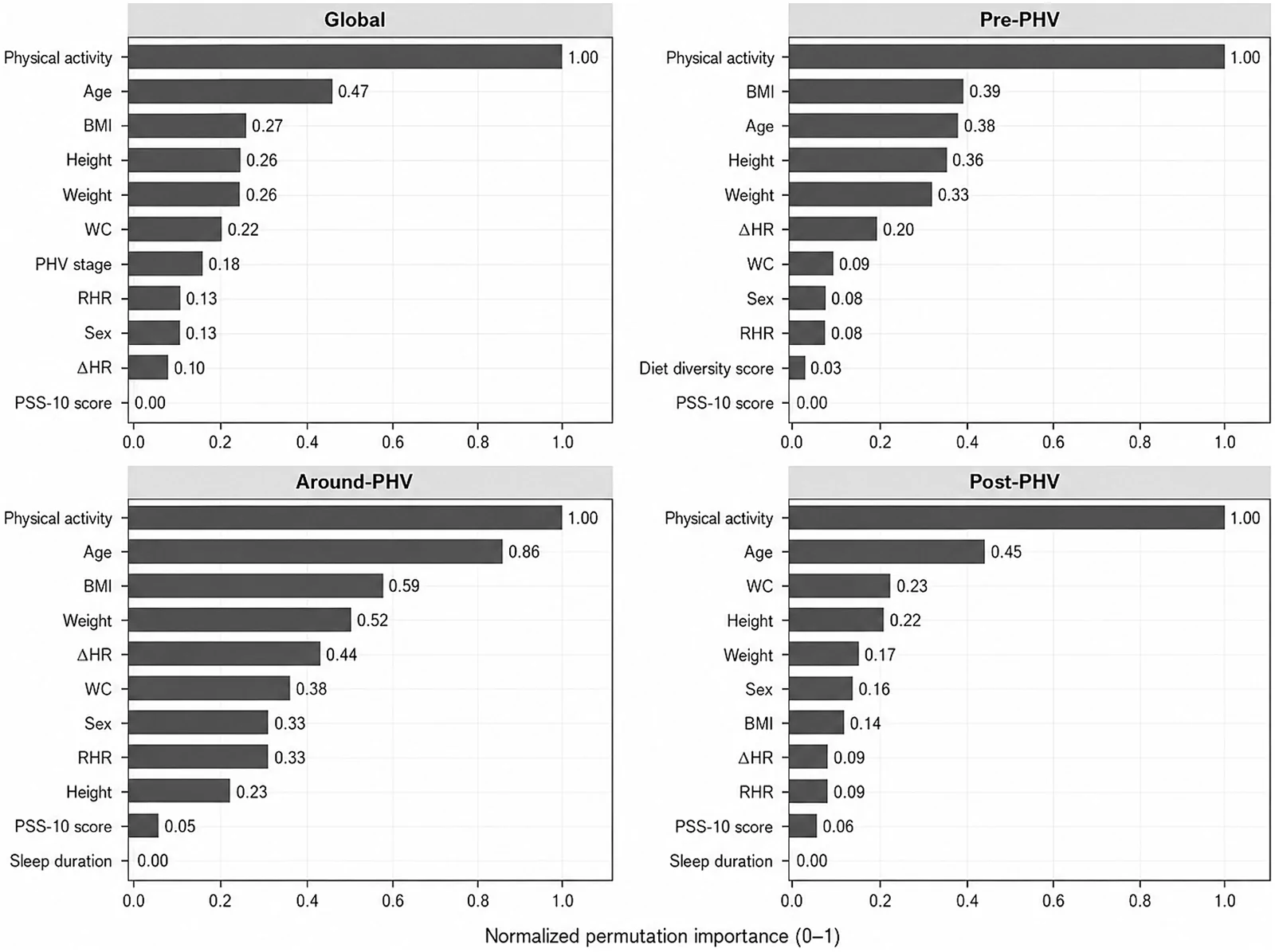
Normalized permutation importance of predictors of 6MWT distance in the global and PHV-specific random forest models. 6MWT, six-minute walk test; PHV, peak height velocity; BMI, body mass index; WC, waist circumference; RHR, resting heart rate; ΔHR, exercise-induced heart rate response; PSS-10, 10-item Perceived Stress Scale.

Stage-specific analyses revealed a marked reorganization of determinant hierarchies across maturation stages. In pre-PHV adolescents, physical activity also had the highest normalized importance (1.00). BMI (0.39), chronological age (0.38), standing height (0.36), and body mass (0.33) showed moderate relative contributions. Exercise-induced heart rate response had a normalized importance of 0.20, whereas waist circumference, sex, resting heart rate, dietary diversity, and perceived stress made smaller contributions. Around PHV, physical activity remained the highest-ranking predictor (1.00), followed by chronological age (0.86). BMI (0.59), body mass (0.52), exercise-induced heart rate response (0.44), and waist circumference (0.38) also contributed to the model predictions. Sex and resting heart rate each showed a normalized importance of approximately 0.33, whereas standing height, perceived stress, and sleep duration made smaller contributions. In post-PHV adolescents, physical activity again showed the highest relative predictive contribution (1.00), followed by chronological age (0.45). Waist circumference (0.23), standing height (0.22), body mass (0.17), sex (0.16), and BMI (0.14) showed smaller contributions. Exercise-induced heart rate response, resting heart rate, perceived stress, and sleep duration contributed comparatively little to the model.

### 3.4 SHAP analyses of stage-specific model contributions to 6MWT performance

Figure 2 presents SHAP summary (beeswarm) plots across PHV stages. In the pre-PHV stage, physical activity showed the highest mean absolute SHAP contribution (approximately 19.2 m), followed by BMI (approximately 9.5 m), chronological age (approximately 7.7 m), and standing height (approximately 7.3 m). During the around-PHV stage, physical activity showed the largest model contribution (approximately 24.4 m), closely followed by chronological age (approximately 22.9 m), with BMI (approximately 10.1 m) and RHR (approximately 9.0 m) also contributing to the model predictions. In the post-PHV stage, physical activity had the largest mean absolute SHAP contribution (approximately 45.3 m), followed by chronological age (approximately 25.9 m), while secondary contributions were observed for standing height (approximately 11.4 m), perceived stress (approximately 8.9 m), waist circumference (approximately 7.7 m), RHR (approximately 7.1 m), body mass (approximately 7.0 m), BMI (approximately 6.9 m), and ΔHR (approximately 5.1 m). Overall, SHAP results showed that physical activity was the leading stage-specific contributor across all PHV stages, while chronological age remained particularly influential around-PHV and post-PHV. Anthropometric, psychosocial, and cardiovascular variables showed smaller and stage-specific contributions. These SHAP values describe model predictions and should not be interpreted as causal effects.

**Figure 2:**
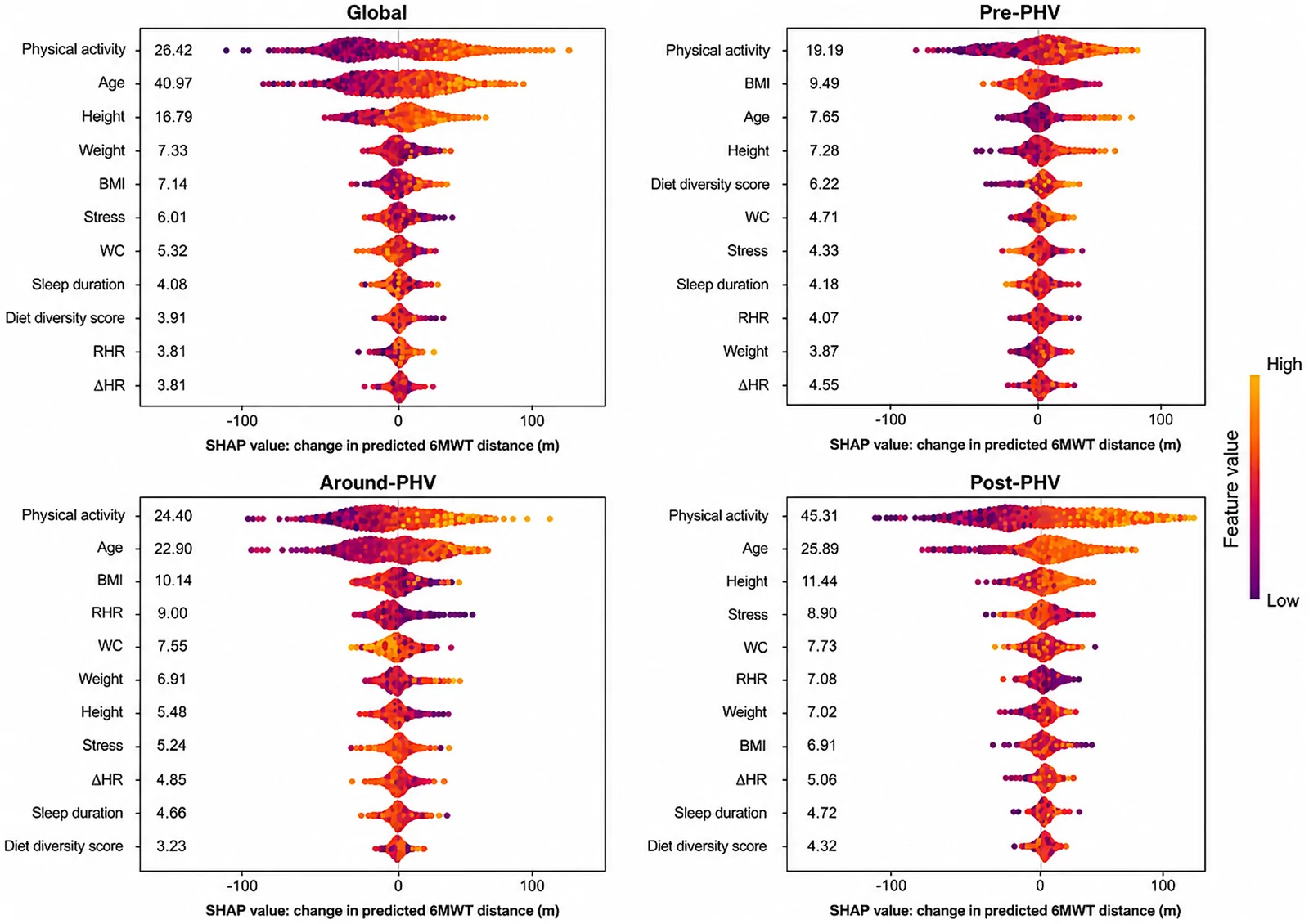
SHAP-based contributions of predictors to 6MWT distance in the global and PHV-specific random forest models. The value printed beside each predictor is its mean absolute SHAP value, in meters. SHAP, Shapley Additive Explanations; 6MWT, six-minute walk test; PHV, peak height velocity; BMI, body mass index; WC, waist circumference; RHR, resting heart rate; ΔHR, exercise-induced heart rate response; Stress, 10-item Perceived Stress Scale score.

### 3.5 Exploratory identification of functional and cardiovascular profiles

The gap statistic reached its maximum at k = 1, indicating that 6MWT performance and cardiovascular responses were primarily distributed along a continuum rather than forming naturally distinct categories. Among the non-trivial solutions examined, the average silhouette width was highest for k = 2 (0.309). Accordingly, the two-cluster solution was retained solely as an exploratory and descriptive representation of functional-cardiovascular variability rather than as evidence of distinct biological states. The global solution classified 1,743 participants into a higher-response profile and 1,423 into a lower-response profile (Figure 3).

**Figure 3:**
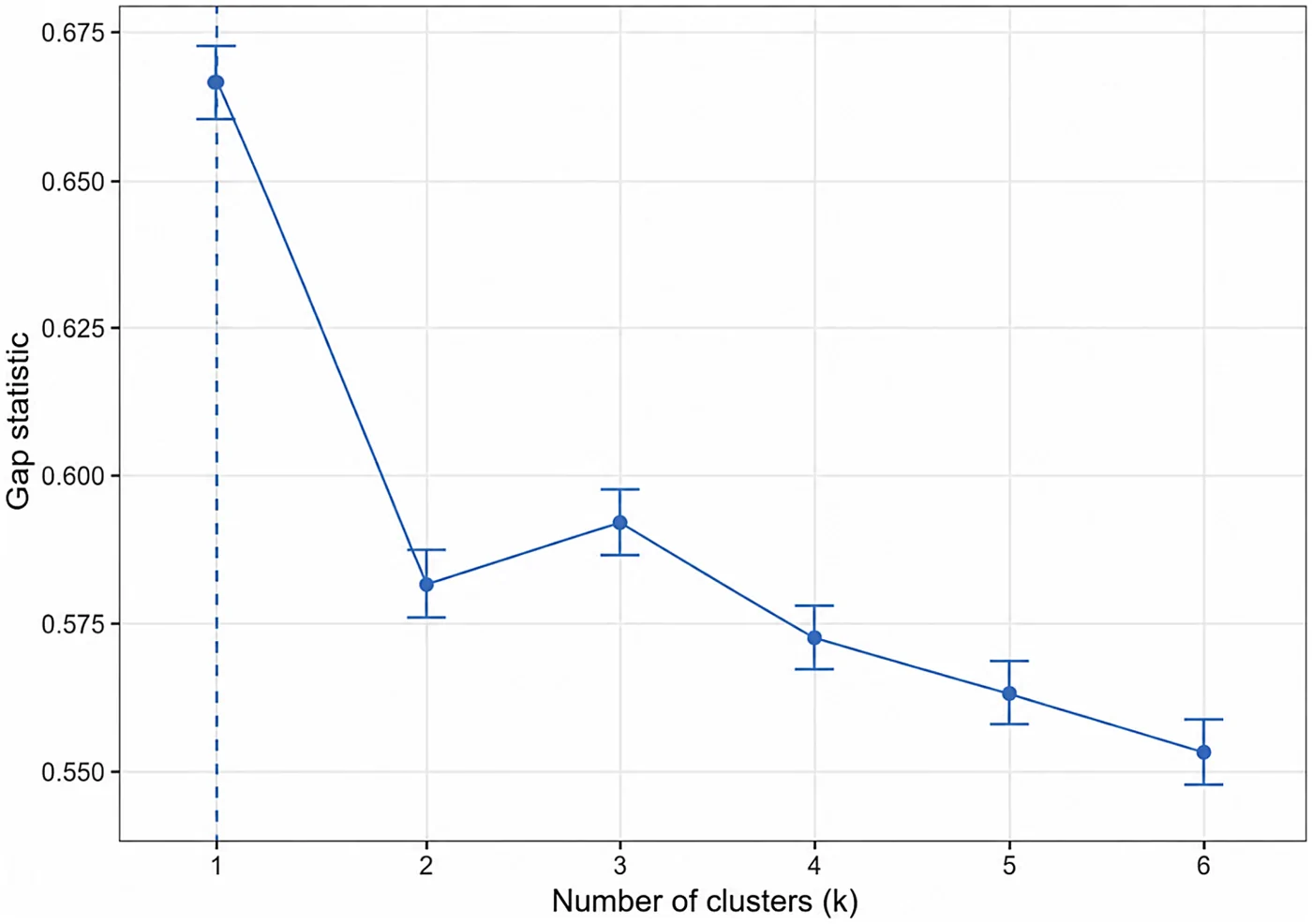
Evaluation of the number of exploratory functional-cardiovascular profiles using the gap statistic. The dashed vertical line marks the solution with the highest gap statistic (k = 1). k, number of clusters.

Figure 4 illustrates a partial separation between lower-response and higher-response functional-cardiovascular profiles across all PHV stages. The first principal component, accounting for 56.4% of total variance, consistently represented the main axis differentiating the two profiles, indicating that 6MWT performance and cardiovascular response formed the dominant source of variability. The second component, accounting for 30.5% of total variance, captured secondary interindividual variability without substantially altering the overall profile distribution. Across pre-PHV, around-PHV, and post-PHV stages, the general direction of profile separation was comparable, although noticeable overlap remained between the 95% confidence ellipses. These findings suggest that the globally derived lower-response and higher-response profiles provide an exploratory descriptive representation of functional-cardiovascular variability across PHV stages; however, they should not be interpreted as robust biological phenotypes or as evidence of within-individual developmental stability throughout puberty.

**Figure 4:**
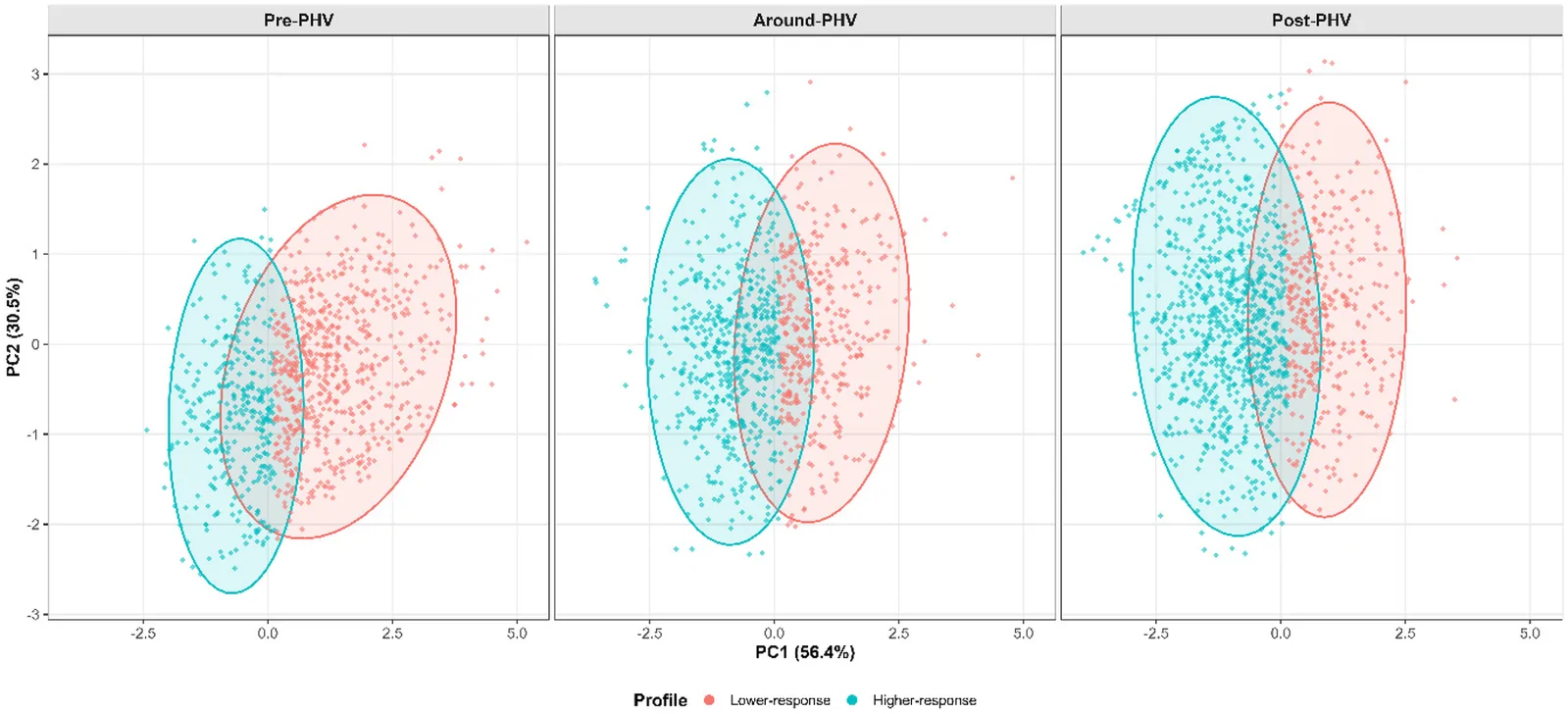
PCA visualization of globally derived exploratory functional-cardiovascular profiles across PHV stages. Each point is one participant and the ellipses are 95% confidence ellipses for each profile. PCA, principal component analysis; PHV, peak height velocity; PC, principal component.

### 3.6 Functional and cardiovascular characteristics of lower- and higher-response profiles

Table 3 presents the functional and cardiovascular characteristics of the globally derived lower-response and higher-response profiles across PHV stages. Across the entire sample, the higher-response profile demonstrated a more favorable functional-cardiovascular pattern, characterized by a greater 6MWT distance (598.97 versus 490.36 m), a lower RHR (61.50 versus 76.67 bpm), and a markedly greater ΔHR (91.32 versus 68.36 bpm) compared with the lower-response profile. This descriptive contrast was preserved across all PHV stages.

**Table 3:** Mean functional and cardiovascular characteristics of the globally derived exploratory profiles, overall and according to PHV stage.

PHV stage	Profile	*n*	6MWT distance (m)	RHR (bpm)	ΔHR (bpm)
Overall	Lower-response	1,423	490.36 ± 132.10	76.67 ± 8.57	68.36 ± 12.59
	Higher-response	1,743	598.97 ± 157.12	61.50 ± 5.92	91.32 ± 13.34
Pre-PHV	Lower-response	630	439.92 ± 110.39	79.36 ± 9.21	69.09 ± 13.84
	Higher-response	329	475.18 ± 117.93	63.47 ± 5.66	94.48 ± 11.09
Around-PHV	Lower-response	401	512.20 ± 124.57	74.63 ± 7.68	68.77 ± 11.67
	Higher-response	552	587.35 ± 132.31	61.80 ± 5.67	91.10 ± 13.11
Post-PHV	Lower-response	392	549.08 ± 141.19	74.41 ± 7.03	66.76 ± 11.19
	Higher-response	862	653.66 ± 156.55	60.56 ± 5.97	90.25 ± 14.08

Values are presented as mean ± SD. ΔHR represents the exercise-induced heart rate response calculated as peak heart rate minus resting heart rate. Profiles were derived from a single global k-means clustering analysis based on standardized 6MWT distance, resting heart rate, and ΔHR.

In pre-PHV, participants assigned to the higher-response profile already exhibited greater cardiovascular responsiveness (ΔHR: 94.48 versus 69.09 bpm), despite a smaller absolute difference in walking distance (475.18 versus 439.92 m; approximately 35 m). During the around-PHV period, the difference in 6MWT distance increased to approximately 75 m (587.35 versus 512.20 m), while the difference in ΔHR remained evident (91.10 versus 68.77 bpm). In post-PHV, the higher-response profile showed the largest absolute performance difference, of approximately 105 m (653.66 versus 549.08 m), together with a lower RHR (60.56 versus 74.41 bpm) and a greater ΔHR (90.25 versus 66.76 bpm). Overall, these patterns indicate that the globally derived profiles maintained comparable functional and cardiovascular contrasts across PHV stages, although their magnitude varied with biological maturation. However, they should be interpreted as exploratory descriptions of continuous functional-cardiovascular variability rather than as stable or distinct physiological phenotypes**.**

## 4 Discussion

This study examined stage-specific prediction patterns for 6MWT performance using supervised and unsupervised machine learning approaches in a large cohort of Tunisian adolescents. Three main findings emerged. First, the predictive performance of RF models differed across PHV stages: it was very limited pre-PHV, modest around-PHV, and highest post-PHV, although a substantial proportion of variance remained unexplained in all stage-specific models. Second, the relative hierarchy of predictive contributors varied across puberty; however, physical activity remained the leading contributor across all PHV stages, whereas chronological age showed its strongest relative contribution during the around-PHV period. Third, exploratory unsupervised analyses identified two lower-response and higher-response functional-cardiovascular profiles across PHV stages, but these should be interpreted as data-driven clusters rather than stable biological states.

### 4.1 Maturation-stage differences in predictive performance for 6MWT distance

One of the main findings of this study is that the predictive performance of multivariate models for 6MWT distance differed across PHV stages. In pre-PHV adolescents, RF models explained only a small proportion of the variance in 6MWT performance, whereas explanatory performance was modest around-PHV and highest post-PHV. The overall pattern is consistent with previous evidence showing that the associations between anthropometric, behavioral, and physiological factors and aerobic fitness variables may differ according to biological maturation status (58–60).

Pre-PHV, adolescents may exhibit substantial biological heterogeneity, with imperfect correspondence between chronological age and physiological development (26, 80). Therefore, 6MWT performance during this phase may be influenced by unmeasured developmental variability, neuromotor control, movement economy, and growth-related coordination differences, thereby limiting model performance (17, 26). Around-PHV, rapid somatic growth and hormonal changes may introduce additional heterogeneity, which could partly explain the modest predictive performance observed in this phase (61, 62). In contrast, post-PHV participants may show more consistent group-level associations among body structure, habitual physical activity, cardiovascular regulation, and 6MWT performance (63, 64), which may partly explain the comparatively better predictive performance of the post-PHV model. However, this model still accounted for only a limited proportion of the total variability in 6MWT distance, indicating that additional physiological, behavioral, environmental, and measurement-related factors were not captured by the available predictors.

These results highlight the limitations of age-based prediction models at the onset of adolescence and show the importance of explicitly considering biological maturation when assessing adolescent 6MWT performance. Potentially relevant unmeasured factors include stroke volume, cardiac output, hemoglobin-related oxygen transport, skeletal-muscle oxygen extraction, mitochondrial adaptations, and muscle oxidative capacity. However, these mechanisms were not directly assessed in the present study. From a practical perspective, prediction and interpretation of 6MWT performance should therefore remain cautious across all PHV stages, particularly pre-PHV and around-PHV.

### 4.2 Stage-specific model contributions to 6MWT performance

Beyond changes in model performance, this study revealed maturation-related variation in the relative hierarchy of predictive contributors. Physical activity remained the leading contributor to 6MWT performance across all PHV stages, whereas chronological age gained greater relative importance during the around-PHV period, where it ranked as the second after physical activity.

Pre-PHV, the predominance of physical activity suggests that behavioral factors may play an important role in functional performance despite ongoing growth and developmental variability. This observation is consistent with previous studies showing that regular physical activity is strongly associated with cardiorespiratory fitness in children, regardless of size or maturation stage (65, 66). Regular physical activity may promote central and peripheral adaptations, including greater stroke volume, improved skeletal-muscle capillarization, enhanced mitochondrial density and oxidative enzyme activity, and greater peripheral oxygen extraction (67–69). These adaptations may improve walking economy and the capacity to sustain submaximal exercise.

Around-PHV, this pattern may have a developmental basis. Although chronological age is not a direct measure of biological maturation, small age differences during this transitional period may coincide with substantial variation in hormonal exposure, growth velocity, cardiac dimensions, muscle mass, and neuromuscular development (7, 10, 62). Maturation-related increases in stroke volume, oxygen-delivery capacity, mitochondrial function, and skeletal-muscle oxygen extraction may also vary substantially between adolescents of similar chronological age (70–73). Previous longitudinal studies have shown that aerobic capacity and physical performance may improve rapidly during the peripubertal period, with differences in maturation timing contributing to interindividual variability in performance (62, 74, 75). Accordingly, the greater contribution of chronological age around-PHV may reflect the heterogeneous developmental changes occurring during this stage rather than a reduced influence of physical activity.

Post-PHV, physical activity remained the leading predictive contributor, exceeding the relative contributions of chronological age and anthropometric variables. This finding suggests that, once the most rapid phase of somatic growth has passed, interindividual differences in 6MWT performance may be more strongly associated with habitual activity and physical conditioning than with maturation-related variation (76). Repeated activity exposure may support the maintenance or further development of stroke volume, muscular oxidative capacity, mitochondrial function, and oxygen extraction, thereby contributing to improved submaximal performance (67). However, these mechanisms require direct physiological confirmation. From a translational perspective, the findings emphasize physical activity as the principal modifiable predictive contributor across all PHV stages, while highlighting the need to account for age-related developmental variability around-PHV.

### 4.3 Stage-specific roles of anthropometric and physiological factors

Anthropometric variables showed secondary and maturation-specific predictive contributions to 6MWT performance. In pre-PHV participants, BMI and standing height contributed to the model, although their influence remained substantially lower than that of physical activity. These associations are consistent with previous studies suggesting that body size may influence walking performance through stride length, locomotor efficiency, and the energetic cost of movement (77, 78). Around-PHV, BMI, body mass, waist circumference, and standing height showed modest contributions, potentially reflecting the heterogeneous somatic changes. Variability in growth velocity and body composition during this transitional period may temporarily influence gait mechanics, coordination, and movement efficiency, thereby contributing to interindividual differences in functional performance (79, 80). Post-PHV standing height and waist circumference remained secondary contributors, while the relative influence of most anthropometric variables was limited compared with physical activity and chronological age. Waist circumference may partly reflect central adiposity, which has been associated with reduced cardiorespiratory fitness and functional performance in adolescents (81, 82). Overall, the findings suggest that anthropometric characteristics provide complementary rather than dominant predictive information across maturation stages.

Cardiovascular variables, including RHR and ΔHR, generally showed modest contributions in supervised models. This interpretation is consistent with previous studies indicating that cardiovascular indices are not dominant predictors of submaximal functional performance when anthropometric and behavioral factors are considered simultaneously (83, 84). In the unsupervised analysis, RHR and ΔHR contributed directly to the definition of the exploratory lower-response and higher-response profiles because they were included as clustering variables. Their role in distinguishing the profiles should therefore be interpreted as a descriptive feature of the clustering solution rather than as independent evidence that they are primary physiological determinants of performance.

### 4.4 Exploratory functional and cardiovascular profiles across PHV stages

Unsupervised clustering provided an exploratory two-profile representation of functional-cardiovascular variability, corresponding to lower-response and higher-response profiles. However, the gap statistic favored a single-cluster solution, and the average silhouette width for k = 2 was modest (0.309), indicating that the underlying data were primarily continuous rather than naturally divided into distinct categories. The profiles were derived once in the complete sample and subsequently examined across PHV stages; therefore, their presence within pre-PHV, around-PHV, and post-PHV groups should not be interpreted as evidence of developmental stability or within-individual tracking throughout puberty.

The higher-response profile was characterized by a longer 6MWT distance, a lower RHR, and a greater ΔHR than the lower-response profile. These descriptive contrasts were observed within each PHV stage, although their magnitude varied across maturation categories. Previous studies have reported moderate-to-strong tracking of cardiorespiratory fitness and autonomic cardiovascular characteristics across childhood and adolescence, suggesting that some aspects of functional and physiological status may persist over time (74, 85, 86). Nevertheless, because 6MWT distance, RHR, and ΔHR were used directly to construct the clusters in the present study, the observed differences are characteristics of the clustering solution itself rather than independent evidence of greater cardiovascular efficiency or superior physiological adaptation. The cross-sectional design does not allow conclusions regarding the tracking, persistence, or within-individual stability of these profiles throughout puberty.

From a clinical and public health perspective, the lower-response profile may help describe adolescents presenting a combination of shorter walking distance, higher RHR, and a smaller heart rate response. Previous evidence has linked low cardiorespiratory fitness and unfavorable cardiovascular profiles in youth with increased cardiometabolic risk and has emphasized the potential value of early physical activity interventions (87, 88). However, the present profiles have not been validated as clinical risk categories or screening tools. Longitudinal studies incorporating direct physiological and clinical outcomes are needed to establish their reproducibility and prognostic relevance.

### 4.5 Methodological implications of explainable machine learning

One of the main methodological contributions of this study is the use of explainable machine learning approaches to characterize maturation-specific predictive contributors to 6MWT performance. Permutation importance and SHAP provided complementary information on model behavior and the relative contributions of predictors across PHV stages. Permutation importance quantified the relative dependence of model performance on each predictor, whereas SHAP estimated the magnitude and direction of individual-level contributions to model predictions. In particular, mean absolute SHAP values expressed contributions in meters of predicted 6MWT distance, thereby facilitating interpretation of the model outputs beyond conventional measures of predictive accuracy (28, 89). However, because separate models were fitted within each PHV stage, comparisons between stages should be interpreted cautiously and primarily in terms of relative contribution patterns rather than causal or directly equivalent effect sizes.

Explainable machine learning supported established associations, particularly the prominent predictive contribution of physical activity, while also revealing maturation-related variation in the relative contributions of chronological age, anthropometric characteristics, and cardiovascular responses. These approaches may be especially useful for examining heterogeneous adolescent populations in whom growth, maturation, behavior, and physiological responses can interact through complex and potentially nonlinear relationships (90, 91). Nevertheless, variable-importance and SHAP estimates describe how predictors contribute to model predictions and do not establish causality. The present findings therefore support the integration of explainable machine learning into developmental exercise science as a complementary approach to conventional statistical analyses, while emphasizing the need for external validation and longitudinal confirmation.

### 4.6 Limitations and future directions

Several limitations must be acknowledged. The cross-sectional design precludes causal inference and limits understanding of individual developmental trajectories. Longitudinal studies are needed to determine whether the relative hierarchy of predictive contributors and exploratory profile assignments change within individuals during puberty (63, 74). Functional capacity was assessed using a submaximal test; incorporating maximal effort measures could provide additional information (92). Biological maturation was estimated using anthropometric equations rather than direct hormonal assessment, which may have introduced misclassification (53, 93, 94). This anthropometric estimation may also introduce structural dependence with body-size predictors, warranting cautious interpretation of their predictive contributions. Physical activity was self-reported, and may therefore be subject to recall bias (48). In addition, the absence of systematic repeated anthropometric measurements may have introduced residual random measurement variability, although standardized procedures and direct supervision were used to reduce this risk. Finally, because all participants were school-based adolescents from Tunisia, the generalizability of the findings to other geographic, ethnic, socioeconomic, athletic, and clinical populations may be limited.

Future research should include longitudinal designs, objective measures of physical activity, and direct hormonal or physiological markers of biological maturation, and external validation in independent and culturally diverse cohorts. Combining explainable machine learning with direct measurements of oxygen uptake, stroke volume, arteriovenous oxygen difference, and skeletal-muscle oxidative capacity could provide a stronger mechanistic interpretation of 6MWT performance across maturation. Intervention studies are also warranted to determine whether targeted strategies improve 6MWT performance and cardiovascular responses and whether the exploratory functional-cardiovascular profiles are reproducible and responsive to change.

## 5 Conclusion

In summary, 6MWT performance was associated with behavioral, anthropometric, cardiovascular, and maturation-related characteristics in this cross-sectional adolescent sample. Random forest model performance was very limited pre-PHV, modest around-PHV, and comparatively higher post-PHV, although substantial variance remained unexplained across all PHV groups. Physical activity was the leading predictive contributor across all stages, whereas chronological age showed its strongest relative contribution around-PHV. Exploratory clustering described lower-response and higher-response functional-cardiovascular profiles, which should be interpreted as descriptive summaries of continuous variability rather than stable biological or clinical categories. These findings support PHV-sensitive interpretation of adolescent 6MWT performance and require longitudinal and external validation.

## Data Availability

The raw data supporting the conclusions of this article will be made available by the authors, without undue reservation.
